# Persistently High Hip Circumference after Bariatric Surgery Is a Major Hurdle to Successful Hip Replacement

**DOI:** 10.1155/2014/786474

**Published:** 2014-03-04

**Authors:** Menachem M. Meller, Amber B. Courville, Anne E. Sumner

**Affiliations:** ^1^Orthopedics Department, Mercy Philadelphia Hospital, 501 South 54th Street, Philadelphia, PA 19143, USA; ^2^Nutrition Department, Clinical Center, National Institutes of Health (NIH), Bethesda, MD 20892, USA; ^3^Diabetes, Endocrinology and Obesity Branch, NIDDK, NIH, Bethesda, MD 20892, USA

## Abstract

The prevalence of class III obesity (BMI ≥ 40 kg/m^2^) in black women is 18%. As class III obesity leads to hip joint deterioration, black women frequently present for orthopedic care. Weight loss associated with bariatric surgery should lead to enhanced success of hip replacements. However, we present a case of a black woman who underwent Roux-en-Y gastric bypass with the expectation that weight loss would make her a better surgical candidate for hip replacement. Her gastric bypass was successful as her BMI declined from 52.0 kg/m^2^ to 33.7 kg/m^2^. However, her hip circumference after weight loss remained persistently high. Therefore, at surgery the soft tissue tunnel geometry presented major challenges. Tunnel depth and immobility of the soft tissue interfered with retractor placement, tissue reflection, and surgical access to the acetabulum. Therefore a traditional cup placement could not be achieved. Instead, a hemiarthroplasty was performed. After surgery her pain and reliance on external support decreased. But her functional independence never improved. This case demonstrates that a lower BMI after bariatric surgery may improve the metabolic profile and decrease anesthesia risk, but the success of total hip arthroplasties remains problematic if fat mass in the operative field (i.e., high hip circumference) remains high.

## 1. Introduction

According to NHANES 2009-2010, the prevalence of class III obesity (BMI ≥ 40 kg/m^2^) in black, white, Hispanic, and Mexican American women is 18.0%, 7.3%, 6.1%, and 6.7%, respectively [1]. For men, the prevalence also varies by ethnicity but is lower than 8% in all groups [1]. Due to their high rate of obesity, black women are highly likely to come to the attention of orthopedic surgeons after bariatric surgery for hip replacement. Bariatric surgery is associated with many benefits including the eradication or improvement of hyperglycemia, hypertension, dyslipidemia, hepatic steatosis, and obstructive sleep apnea [2]. All of these metabolic changes lead to decreased risk of anesthesia and postsurgical metabolic complications. But in our urban orthopedic clinic, we are observing persistently high hip circumferences in black women, even after major weight loss (MMM). Therefore we frequently encounter an unpublished and unexpected orthopedic risk: inadequate loss of subcutaneous fat in the operative field. To demonstrate the challenges, a representative case is presented.

## 2. Case

With the expectation that her back and hip pain would resolve, a 51-year-old black woman, with a BMI of 52.0 kg/m^2^, underwent laparoscopic Roux-en-Y gastric bypass. Within three years she achieved a BMI of 33.7 kg/m^2^. At this lower BMI, she reported that her back pain had improved but due to persistent severe right hip pain, her ability to ambulate remained compromised. She requested surgical relief.

**Figure 1:**
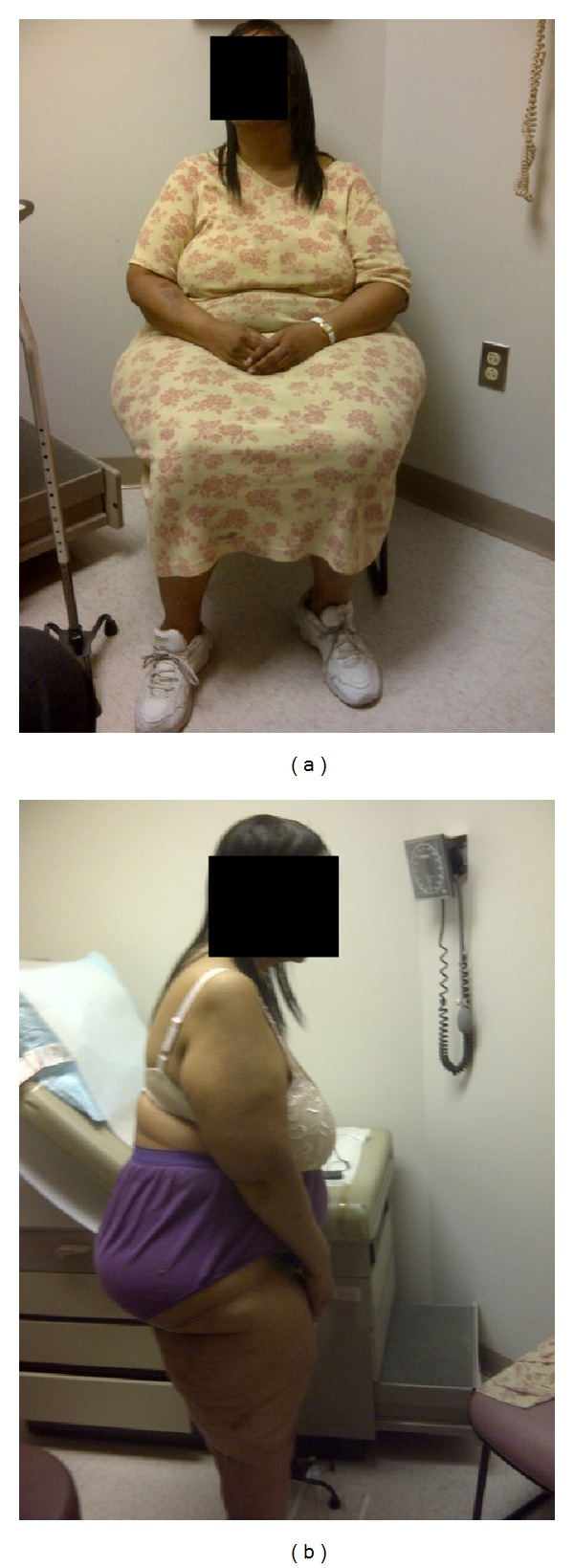
51-year-old black woman with BMI of 42.0 kg/m^2^ after bariatric surgery, waist circumference of 107 cm, and hip circumference of 155 cm. (a) Seated anterior view. (b) Profile standing.

At her preoperative orthopedic evaluation, she had a Harris Hip Score of 17, and even though her BMI was 33.7 kg/m^2^, her hip circumference was proportionately much higher. Preoperative photographs of her with a BMI of 33.7 kg/m^2^ were not taken. Photographs from 3 years later when her BMI had increased to 42.0 kg/m^2^ have been obtained (Figure 1). At the time these photographs were taken, her waist and hip circumferences were 107 cm and 155 cm, respectively. Her overall proportions with a BMI of 42.0 kg/m^2^ were visually similar to her preorthopedic surgery measurements when her BMI was 33.7 kg/m^2^.

Due to her high hip circumference, minimally invasive techniques were not an option. The massive soft tissue mass in the surgical field totally obstructed bony landmarks and precluded both computer and image navigation techniques. She underwent a 4-hour operation during which extensile incisions were utilized to improve access and component positioning. Due to the 15 cm depth of the soft tissue tunnel, combined with the inability to reflect the soft tissue flaps, a traditional cup placement could not be achieved. Therefore, instead of the more optimal total hip replacement, a hemiarthroplasty was performed. Postoperatively, a chronically draining wound which took months to stabilize occurred.

Nonetheless she had transient improvement in hip score. However, her ambulation at 1 and 3 years after hemiarthroplasty was only minimally improved and she is categorized as housebound ambulatory with a Harris Hip Score of 18.

## 3. Discussion

For the woman presented, bariatric surgery was successful in achieving weight loss so that BMI declined from 52.0 kg/m^2^ to 33.7 kg/m^2^. However, her persistently high hip circumference left her with the same technical challenges during hip replacement surgery as if she had not undergone the weight loss intervention. Even with a BMI in the category of class I obese, her body fat distribution was such that a total hip replacement could not be accomplished. Due to the size, shape, and mobility of the soft tissues in the operative field, adequate exposure and access to the acetabular component were not achievable.

**Figure 2:**
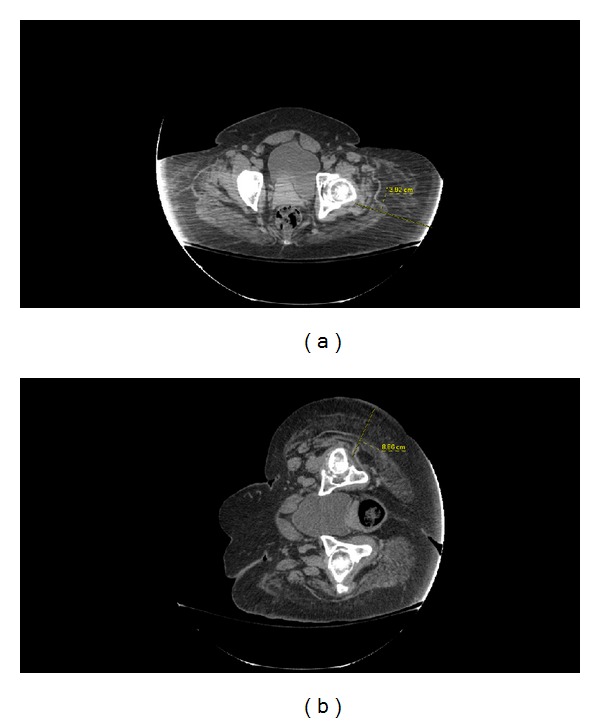
CT imaging to demonstrate soft tissue geometry and gravitational changes with position. (a) Axial supine. (b) Lateral decubitus. These scans are presented as an example and are from a black woman with BMI of 45.0 kg/m^2^.

This case raises awareness that neither absolute weight nor BMI is sufficient to define whether the subsequent orthopedic risks have been minimized. Attention needs to be paid to the mass of fat in the operative field. Importantly, this large mass of subcutaneous fat can be predicted preoperatively by simply measuring hip circumference and performing hip axial imaging by CT (Figure 2). Consistent with our clinical experience, the importance of hip circumference as a way to predict high body fat content has recently been validated. The ratio of hip circumference to height known as the Body Adiposity Index (BAI), has been shown to be superior to BMI as a measure of body fat [3] (Body Adiposity Index = (hip circumference/(height^1.5^)) − 18).

Overall, this case presentation has special relevance for black women. Women have more subcutaneous fat than men and black women have more subcutaneous fat than white women [4, 5]. Therefore even after successful weight loss black women are likely to continue to have a relatively high volume of subcutaneous fat. To optimize orthopedic outcomes related to total hip replacement, surgeons need to evaluate patients independent of BMI for this possibility and plan accordingly. At this time, bariatric and orthopedic surgeons focus on BMI rather than hip circumference.

We bring this issue forth because there appears to be an absence of appreciation of this issue. A literature search led us to the identification of only two articles discussing hip replacement surgery following bariatric surgery [6, 7]. Both of these articles described the benefits of bariatric surgery and neither addressed the orthopedic issues we describe. Because extensive fat in the surgical field is so commonly encountered in our practice, we have developed modifications that have been helpful (MMM) (Table 1). We appreciate the limitations in waist and hip circumference measurements being made 3 years following her hip replacement surgery. However her body mass distribution, including her pear shaped body habitus, was preserved allowing for some of the observations and recommendations we have made. We hope this case presentation will lead to improved outcomes and bring attention to the need to focus not just on BMI but also hip circumference when considering the options for hip arthroplasty.

## Figures and Tables

**Table 1:** Modifications to consider for hip arthroplasty with high hip circumference.

Challenge	Standard procedure	Modifications to consider
Preoperative workup	Hip and pelvis radiographs	Waist and hip circumferences* Preoperative range of motion/flexibility assessment Axial supine and lateral decubitus CT (Figure 2 is presented as an example)
Preoperative setup in surgical suite	“Bean bag” with usual time and staffing	Schedule for oversized 1000-pound table, lateral positioners, oversized instruments, and retractors, and additional staff and operating time and resources
Position on operative table	Approach as per surgeon's preference	Anterolateral approach
Evaluation of tunnel depth	Direct inspection	Intraoperative direct measurements
Visualization	Direct inspection	Improved illumination and retraction
Illumination	Quartz halogen, LED, focused high intensity lighting	Supplemental headlamps, flashlights
Tissue exposure	“Charnley,” Weitlander, angled, and reverse retractors	Oversized “Charnleys,” Burkhalters, and Beckman's oversized angled retractors and oversized instruments
Tissue compression	Careful soft tissue handling	Broad retractor blades, wider exposure, releasing tension in intervals
Tissue reflection	Careful soft tissue handling	Accommodation for hard immobile adipose tissues
Delivering the femur	One surgical assistant	Multiple surgical assistants and soft tissue releases
Alignment	Guidance rods and skeletal landmarks	More deliberate use of “bone hooks,” retractors, and computer assisted guides

*http://www.cdc.gov/nchs/nhanes/nhanes3/anthropometric_videos.htm.

## References

[1] Flegal KM, Carroll D, Kit BK, Ogden CL (2012). Prevalence of obesity and trends in the distribution of body mass index among US adults, 1999–2010. *Journal of the American Medical Association*.

[2] Buchwald H, Avidor Y, Braunwald E (2004). Bariatric surgery: a systematic review and meta-analysis. *Journal of the American Medical Association*.

[3] Bergman RN, Stefanovski D, Buchanan TA (2011). A better index of body adiposity. *Obesity*.

[4] Després J-P, Couillard C, Gagnon J (2000). Race, visceral adipose tissue, plasma lipids, and lipoprotein lipase activity in men and women: the health, risk factors, exercise training, and genetics (HERITAGE) family study. *Arteriosclerosis, Thrombosis, and Vascular Biology*.

[5] Hill JO, Sidney S, Lewis CE, Tolan K, Scherzinger AL, Stamm ER (1999). Racial differences in amounts of visceral adipose tissue in young adults: the CARDIA (coronary artery risk development in young adults) study. *The American Journal of Clinical Nutrition*.

[6] Kulkarni A, Jameson SS, James P, Woodcock S, Muller S, Reed MR (2011). Does bariatric surgery prior to lower limb joint replacement reduce complications?. *Surgeon*.

[7] Parvizi J, Trousdale RT, Sarr MG (2000). Total joint arthroplasty in patients surgically treated for morbid obesity. *Journal of Arthroplasty*.

